# Effects of Climate Change on the Distribution of *Papilio xuthus*

**DOI:** 10.3390/insects16020131

**Published:** 2025-01-29

**Authors:** Quanwei Liu, Zhuoyuan Wang, Danping Xu, Yaqin Peng, Junhao Wu, Zhiqian Liu, Xiushan Li, Zhihang Zhuo

**Affiliations:** 1 College of Life Science, China West Normal University, Nanchong 637002, China; quanwei66977@foxmail.com (Q.L.); zhuoyuan0620@163.com (Z.W.); danpingxu@foxmail.com (D.X.); pengyaqin2023@foxmail.com (Y.P.); wujunhao0824@gmail.com (J.W.); qnhtvxhp319123@foxmail.com (Z.L.); xiushanli@vip.163.com (X.L.); 2 Medical College, Nanchong Vocational College of Science and Technology, Nanchong 637200, China

**Keywords:** *Papilio xuthus*, MaxEnt, climate change, potential suitable distribution, environmental variables

## Abstract

The *Papilio xuthus* is a widely distributed species in the genus *Papilio* of the family Papilionidae, possessing ecological, ornamental, and socio-economic service values. To determine the ecological role of *P. xuthus* and assess its population distribution under future climate change scenarios, this study utilized the MaxEnt model to predict the geographic distribution of *P. xuthus* in the future and evaluate its population dynamics. The results indicated that *P. xuthus* is currently widely distributed in East Asia, with a high suitability area of 1827.83 × 10^3^ km^2^, primarily in China, Japan, North Korea, and South Korea. Climate change has a significant impact on the geographic distribution of *P. xuthus*, with its high suitability areas decreasing in the future, particularly within China, where the change is projected to be as high as 46.46% under the SSP126 scenario by the 2050s. The centroid of its high-suitability area is expected to shift northeastward. Key environmental variable analysis revealed that Temperature Seasonality, Mean Temperature of the Wettest Quarter, Precipitation of the Wettest Month, and Precipitation of the Warmest Quarter are critical factors influencing the selection of suitable habitats by *P. xuthus*. This study assessed the distribution of *P. xuthus* and provided conservation recommendations, offering a reference for future population control and conservation efforts.

## Simple Summary

The *Papilio xuthus*, belonging to the family Papilionidae, possesses significant ecological, pollination, and ornamental value. To assess future risks and impacts, this study utilized the MaxEnt model to predict its population distribution. The results indicated that *P. xuthus* currently has a broad suitable habitat across East Asia, but the high-suitability areas are likely to experience a significant decline in the future, with the main reduction areas located within China. This provides a valuable reference for future biodiversity conservation in ecosystems.

## 1. Introduction

Under the premise of global climate and environmental change, the reduction in global biodiversity is one of the challenges facing the world [1]. Research indicates that insects, in particular, play a pivotal role in the overall decline, with a higher proportion of insect species facing reductions compared to other taxa [2,3]. Moreover, populations of some common insect species are also dwindling [4]. Butterflies, as important pollinators in natural ecosystems, contribute significantly to biodiversity and environmental health, and serve as indicators of environmental change [5]. With their remarkable diversity, butterflies are found across the globe and have become a focal point in biodiversity research. China, as a key region for butterfly diversity in Asia, holds profound implications for butterfly studies [6]. Most butterflies are oligophagous insects, closely associated with a limited number of host plants during their larval stages, making them highly susceptible to environmental disturbances [5]. Research showed that climate change affected nutritional relationships, seasonality and phenology, behavior, and distribution patterns of butterfly populations [7,8]. These environmental shifts can lead to detrimental impacts, such as declines in butterfly populations [6], underscoring the critical importance of species conservation efforts.

Understanding species’ responses to climate change is currently a significant focus of research. Climate strongly influences species’ access to water and energy, thereby profoundly affecting their distribution patterns [9,10]. The Intergovernmental Panel on Climate Change (IPCC) highlighted in its Fifth Assessment Report the ongoing warming trend, which poses substantial challenges to species survival [11]. Previous studies have shown that changes in temperature and precipitation directly impacted the suitable habitats for butterflies [12,13]. Understanding the relationship between species and climate often requires the use of Species Distribution Models (SDMs). SDMs simulate species’ geographical distribution and ecological requirements based on known species distribution data and relevant environmental factors [14]. The MaxEnt model is one of the most widely used species distribution models [15,16]. Numerous researchers have previously utilized the MaxEnt model to model species distributions [17,18]. For example, Oklahoma et al. used the MaxEnt model to study the impact of highways on the mortality rate of *Danaus plexippus*, providing conservation recommendations for this species [19]. Westwood et al. employed the MaxEnt model to predict the habitat of *Oarisma poweshiek* [20]. Filazzola et al. investigated the relationship between *Parnassius smintheus* and its host plants using MaxEnt, exploring the interactions between insects and their host plants [21]. Therefore, utilizing the MaxEnt model to analyze species distributions is beneficial for understanding the relationships between species and their environment, or between species themselves.

*Papilio xuthus* belongs to the Papilionidae family within the order Lepidoptera. The larvae of *P. xuthus* are herbivorous, primarily feeding on plants within the Rutaceae family, including species of Zanthoxylum and Citrus, thus categorizing them as typical oligophagous insects [22,23]. Given their significant roles in pollination and their esthetic value, they also hold importance for urban gardens. For biodiversity conservation efforts, it is crucial to assess the population distribution of *P. xuthus*, understanding potential impacts on Rutaceae plants in the future or threats faced by its populations. Current research on *P. xuthus* has primarily focused on the molecular level [24,25,26], with limited attention to its distribution areas. Evaluating the distribution and diversity of wild populations of *P. xuthus* butterflies, and predicting their potential distribution, is essential. This study aimed to enhance the understanding of the relationships between climate, species, and nutritional interactions, facilitating the assessment of how the distribution of *P. xuthus* butterflies would change under future climate conditions. This study evaluated the impact of various environmental parameters on species distribution, explored current and future habitat suitability, and examined the relationship between environmental variables and the species. This approach aims to select key conservation areas for *P. xuthus*. The study seeks to provide insights for effective management, conservation, and diversity control of butterfly populations.

## 2. Materials and Methods

### 2.1. Species Occurrence Records

A large and accurate dataset of species distribution served as the basis for constructing the Maxent model [27]. Data on the distribution of *P. xuthus* were mainly obtained from three sources: (1) Global Biodiversity Information Facility (https://www.gbif.org/, accessed on 27 March 2024, coordinates are provided in Table S1); (2) National Specimen Information Infrastructure (NSII) of China (http://www.nsii.org.cn/, accessed on 27 March 2024); (3) Keyword literature searches were conducted to obtain occurrence records, followed by using Google Maps (http://ditu.google.cn/, accessed on 27 March 2024) to determine their longitude and latitude coordinates. After obtaining the coordinate points, spatial filtering was performed using ENMTools v1.4 to reduce data redundancy and the impact of environmental variable covariance in the model. Only one distribution data point was retained within each 10 km × 10 km grid cell [28,29]. Ultimately, the 735 distribution points of *P. xuthus* obtained were incorporated into the model for construction.

### 2.2. Environment Variables

Based on the distribution characteristics of *P. xuthus* and its host plants, altitude (elevation), aspect, and slope variables from the Resource and Environment Science and Data Center of the Institute of Geographic Sciences and Natural Resources Research, Chinese Academy of Sciences (https://www.resdc.cn/, accessed on 27 March 2024), as well as 19 bioclimatic variables from the Global Climate Database (https://worldclim.org/, accessed on 27 March 2024), were selected for MaxEnt modeling (Table S1). During the modeling process, the contribution rates of the 19 environmental variables were first calculated using MaxEnt (Table S1) to eliminate multicollinearity among the bioclimatic variables and retain those with higher contribution rates. Five environmental variables were selected to build the *P. xuthus* prediction model (Table 1). Pearson’s correlation analysis was then performed on the remaining variables using SPSS 25.0 software to exclude mutual influences between environmental factors (Table S2). Environmental factors with absolute correlation coefficients greater than or equal to 0.8 were excluded, and the remaining environmental factors were analyzed for their impact on the distribution of *P. xuthus* [30].

To predict the potential impact of future climate conditions on the distribution of *P. xuthus* and its host plants, the modeling was based on future climate scenarios provided by the Intergovernmental Panel on Climate Change (IPCC) Sixth Assessment Report [31], using the shared socioeconomic pathways (SSPs) SSP126, SSP370, and SSP585 for future scenario modeling. SSP126 represents sustainable development, SSP370 represents regional rivalry, and SSP585 represents development based on fossil fuels or business-as-usual scenarios [32].

### 2.3. Modeling Process and Statistical Analysis

In the Maxent model, the importance of climate variables was assessed in the final model primarily through contribution percentage and ranking importance. Distribution points of the *P. xuthus*, along with relevant environmental data, were imported into Maxent to establish the initial model. During the model calculation process, 75% of the distribution data were used for training the model, while the remaining 25% were used to validate the Maxent model. Cross-validation was used to assess the correlation of important environmental variables, followed by the establishment of environmental response curves to understand the logical relationship between species occurrence probability and environmental factors.

### 2.4. Model Evaluation

The model’s predictive accuracy was evaluated using receiver operating characteristic (ROC) curves and default parameter values. The predictive performance metric was judged based on the area under the ROC curve (AUC). The predictive accuracy of the model was directly proportional to the AUC value; the closer the AUC value was to 1, the better the predictive performance of the model [33]. ArcGIS software was used to extract the prediction data generated by MaxEnt. Based on the model-generated probabilities of occurrence, a thorough study was conducted on the climatic suitability of the *P. xuthus*. By analyzing the modeling results and reviewing previous studies on species from the same family, the distribution of suitable habitats was classified into four levels: 0–0.1 as unsuitable, 0.1–0.3 as low suitability, 0.3–0.5 as moderate suitability, and 0.5–1 as high suitability [34,35]. By inputting a folder containing current and future binary SDMs into the SDMtool v1.4 software, the geometric centers of the current and future high-suitability areas were calculated to analyze the centroid shift in these high-suitability areas [36].

## 3. Results

### 3.1. Model Performance and Variable Selection

**Figure 1:**
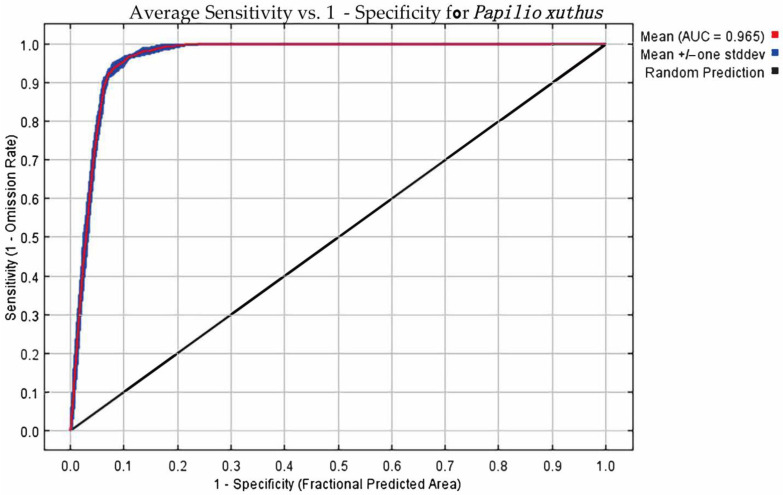
The Receiver Operating Characteristic (ROC) curve and the Area Under the Curve (AUC) value for the study period (1950–2000).

**Figure 2:**
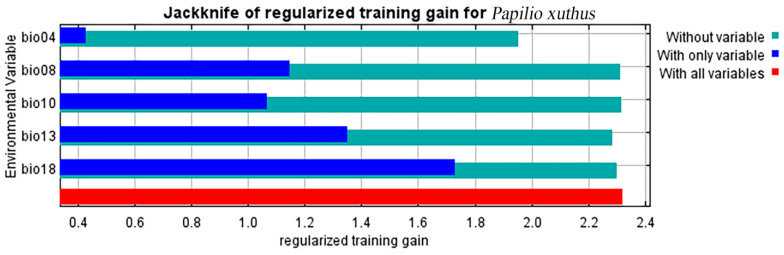
Variable importance, as determined via the folding jackknife test, for *P. xuthus*.

After ten trials, the AUC training value and AUC test value for *P. xuthus* were both 0.965 (Figure 1, Table S3), with the training value being slightly higher. The MaxEnt model demonstrated good predictive performance for the distribution of *P. xuthus* and its host plants. After selection, the five bioclimatic variables involved in modeling *P. xuthus* and their contribution rates (Table S4) were: Precipitation of the Warmest Quarter (70.3%), Temperature Seasonality (18.9%), Precipitation of the Wettest Month (8.8%), Mean Temperature of the Wettest Quarter (1%), and Mean Temperature of the Warmest Quarter (1%). The importance of environmental factors was analyzed using the threshold method (Figure 2, Table S4), with the following importance values: Precipitation of the Wettest Month (47.8%), Mean Temperature of the Wettest Quarter (23.7%), Temperature Seasonality (15.4%), Precipitation of the Warmest Quarter (7.5%), and Mean Temperature of the Warmest Quarter (10%). Given that the model needs to consider the most influential factors based on contribution rates [37] and correlation analysis, only bio04, bio08, bio13, and bio14 were analyzed for their impact on *P. xuthus*.

### 3.2. Environmental Variables Affecting the Geographical Distribution of P. xuthus

**Figure 3:**
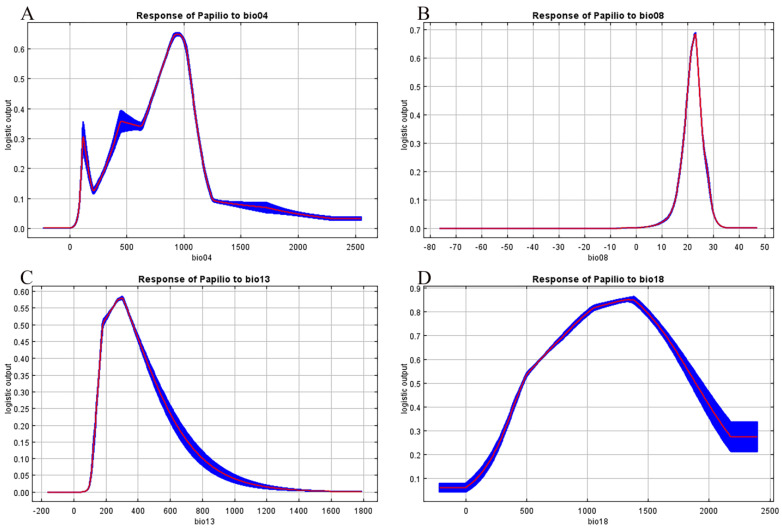
Response curve of environmental variables to the distribution probability of *P. xuthus*. Among them, (**A**) represents Temperature Seasonality (bio04), (**B**) represents Mean Temperature of the Wettest Quarter (bio08), (**C**) represents Precipitation of the Wettest Month (bio13), and (**D**) represents Precipitation of the Warmest Quarter (bio18). The blue areas in the figure represent the environmental response range of the ten models, and the red curve represents the average value.

Based on the response curves of environmental variables to the distribution probability of *P. xuthus* in the MaxEnt model (Figure 3A–D), the suitable environmental ranges for the species’ potential distribution were determined, as detailed in Table 1. The suitable ranges (optimal values) for the environmental factors were as follows: Temperature Seasonality ranged from 773.35 to 1054.28 °C (941.72 °C), mean temperature of the Wettest Quarter ranged from 20.11 to 24.42 °C (22.92 °C), precipitation of the Wettest Month ranged from 179.05 to 368.74 mm (299.85 mm), and precipitation of the Warmest Quarter ranged from 466.25 to 1883.96 mm (1342.09 mm). The distribution probability of *P. xuthus* was limited under conditions where these environmental factors were either too high or too low, indicating that the species is suited to more temperate environments (Table 2).

### 3.3. Potential Distribution of P. xuthus in the Current Period

**Figure 4:**
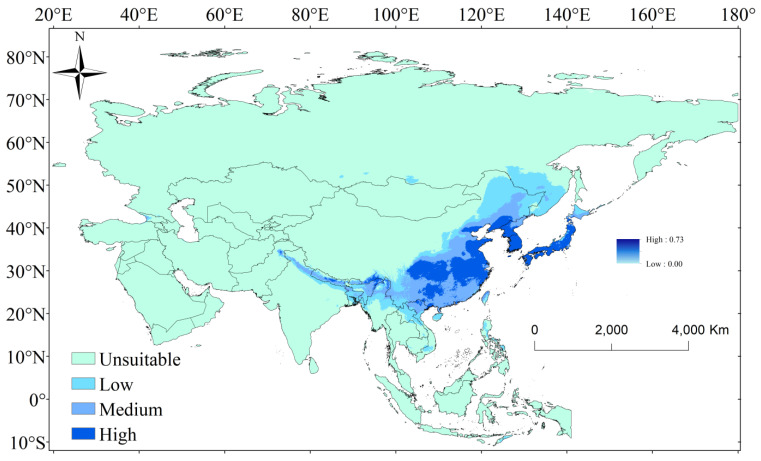
Status of the suitable climatic distribution of *P. xuthus*. The shades of blue from light to dark represent unsuitable, low suitability, medium suitability, and high suitability, respectively.

The distribution map of the most suitable habitat for *P. xuthus* was constructed using the MaxEnt model (Figure 4). Under current climatic conditions, the suitable habitat of *P. xuthus* is primarily distributed across East Asia, with populations extending from south to north, specifically from Vietnam and Laos in the south to Russia in the north. High-suitability areas were concentrated in the middle and lower reaches of the Yangtze River in China, as well as in North Korea, South Korea, and Japan, with a total area of 1827.83 × 10^3^ km^2^ (Table 3). Low-to-medium suitability areas were widespread in China, while medium suitability areas were sparsely found in countries such as India, Pakistan, and Japan, with a total area of 2147.53 × 10^3^ km^2^ (Table 3). Low-suitability areas were also sparsely found in countries such as Russia and Myanmar, with a total area of 2741.01 × 10^3^ km^2^ (Table 3).

### 3.4. Potential Distribution of P. xuthus in the Future Period

**Figure 5:**
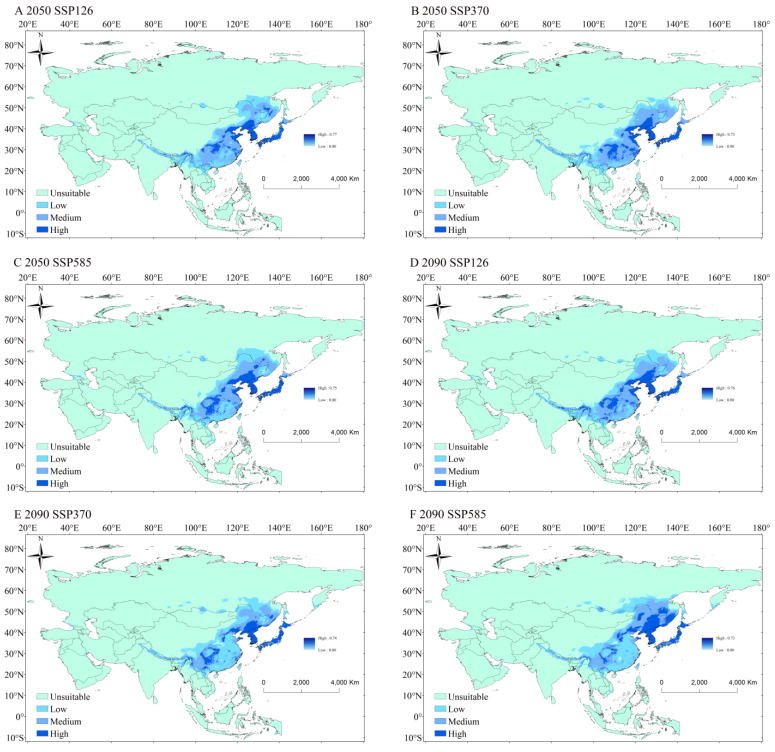
The potential distribution of *P. xuthus* in suitable regions under different climatic conditions. The shades of blue from light to dark represent unsuitable, low suitability, medium suitability, and high suitability, respectively.

According to the prediction results under the three climate change scenarios SSP126, SSP370, and SSP585, the suitable habitat areas for *P. xuthus* in the 2050s and 2090s are shown in Figure 5, and the areas are presented in Table 3. In the future, the high-suitability areas of *P. xuthus* were predicted to migrate toward higher altitudes and show a decreasing trend. The reduction was not significant in Japan, North Korea, and South Korea, but was extremely pronounced in China. In particular, the middle and lower reaches of the Yangtze River basin saw many areas shift to medium/low suitability, leading to an overall increase in the area of medium/low suitability. In the 2050s under the SSP585 scenario, the high-suitability area was the most widespread, primarily distributed in northern China, Japan, North Korea, and South Korea, with a total area of 1531.39 × 10^3^ km^2^. The distribution of high-suitability areas in the 2090s was similar to that in the 2050s, with the SSP370 scenario showing the lowest distribution of high-suitability areas, covering 1341.44 × 10^3^ km^2^. Under the SSP585 scenario, the high-suitability area for *P. xuthus* was the largest, covering 1742.17 × 10^3^ km^2^, which was close to the current distribution, with a reduction in only 4.69%. Extraction and analysis of the potential future suitable habitat areas of *P. xuthus* in China (Table S5) revealed that all high-suitability areas showed a decrease, with the change being significant. The reduction ranged from 24.85% (SSP126 in the 2090s) to 46.46% (SSP126 in the 2050s). The relative change in total area was greater in Asia.

### 3.5. Shift in the Centroids of Highly Suitable Habitats Under Three Future Climate Scenarios

**Figure 6:**
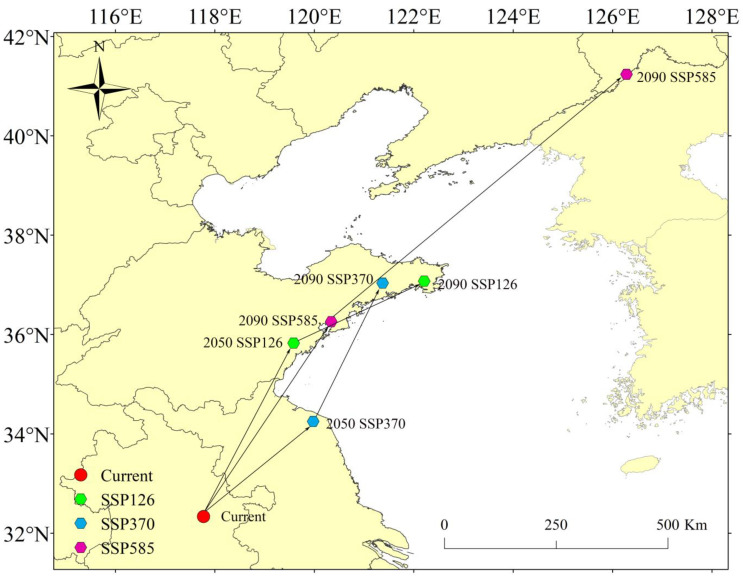
Centroids of highly suitable habitats.

Centroid analysis of the high-suitability areas for *P. xuthus* revealed a significant shift in its centroid, with an overall migration toward the northeast (Figure 6). Under the current scenario, the centroid was located in Suzhou City, Anhui Province, China (32.33° N, 117.77° E). In the future, under the SSP126 scenario, the centroid was predicted to migrate to Yantai City, Shandong Province (37.07° N, 122.21° E) in 2050, and to Jining City, Shandong Province (35.82° N, 119.58° E) in 2090. Under the SSP370 scenario, the centroid first moved to Lianyungang City, Jiangsu Province (34.25° N, 119.97° E), then shifted to Rizhao City, Shandong Province (37.03° N, 121.37° E). Under the SSP585 scenario, the centroid first migrated to Weifang City, Shandong Province (36.25° N, 120.34° E), and later, in the 2090s, moved to Yanbian Korean Autonomous Prefecture, Jilin Province (41.23° N, 126.28° E).

## 4. Discussion

The Maxent model was a commonly used species distribution modeling (SDM) tool for identifying suitable habitats for specific species [38]. In this study, 20 bioclimatic variables were utilized as primary environmental factors, and the Maxent model was applied to simulate the potential suitable distribution areas of *P. xuthus* in China. The model’s accuracy was evaluated using the AUC value, and the results show that the modeling performance for *P. xuthus* is excellent [33]. The model results indicated that *Papilio xuthus* has a wide distribution in China, Japan, North Korea, and South Korea, with nearly all areas in Japan, North Korea, and South Korea being high-suitability areas. In the future, the overall area of high-suitability zones for *P. xuthus* decreased, while the area of medium/low-suitability zones increased. The high-suitability area in the Yangtze River Basin of China significantly decreased, while it increased noticeably in northern China.

An analysis of the key environmental variables affecting the occurrence probability of *P. xuthus* was conducted, and the corresponding response curves were obtained. The results showed that the probability of *P. xuthus* occurrence varied with changes in key environmental factors (Figure 5). In this study, the environmental factors with the highest contribution to the distribution of *P. xuthus* were precipitation and temperature, which aligns with previous conclusions that changes in temperature and precipitation directly affect the suitable habitats for butterflies [12,13]. Precipitation was the main environmental factor influencing the distribution of *P. xuthus*, with a total contribution rate of 79.1%. Drought is one of the important factors affecting insect distribution [39]. Precipitation provides a humid living environment for insects, preventing the harmful effects of drought [40]. However, as precipitation increases, the transmission rate of insect pathogens, such as fungi, also increases [11], reducing the probability of insect occurrence. Additionally, heavy rainfall can suppress insect larval feeding behavior, and prolonged rainfall may cause insects to stop feeding and starve to death [41]. Furthermore, heavy rainfall directly affects the survival of herbivorous insects, as research has shown that it forces insects to leave host plants [42], thereby reducing their distribution due to the relationship between insects and food [42]. In this study, *P. xuthus* was significantly affected by precipitation during the warmest quarter, with a higher probability of occurrence when precipitation ranged from 466.25 to 1883.96 mm (1342.09 mm). Studies have shown that in recent years, extreme rainfall in the middle and lower reaches of the Yangtze River has approached 1800 mm per month, with an increasing trend in precipitation intensity [43]. This has led to a decrease in the potential distribution of *P. xuthus* in that region. Besides precipitation, temperature also influenced *P. xuthus*. Herbivorous insects’ responses to temperature changes may depend on the thermal niche they occupy [44,45]. Insects are highly sensitive to temperature changes in terms of their physiological activity [46], and both excessively low and high temperatures can inhibit their activity, especially when temperatures are too high, as physiological processes may be directly disrupted [47]. In this study, the core environmental factor affecting *P. xuthus* distribution was the mean temperature of the Wettest Quarter, with a higher probability of occurrence when temperatures ranged from 20.11 to 24.42 °C (22.92 °C). Previous studies have shown that *P. xuthus* develops well at temperatures in this range [48]. Research also found that, under the current scenario, the distribution of *P. xuthus* spanned both southern and northern regions, which, respectively, correspond to temperate monsoon climates and subtropical monsoon climates. With future climate warming, southern temperatures are likely to increase, prompting *P. xuthus* to migrate to areas with lower temperatures [49]. The study suggested that *P. xuthus* populations are likely to migrate toward more northern and higher-altitude areas in the future, further confirming that temperature changes significantly impact the population distribution of *P. xuthus*. This study helps in understanding the relationship between the environment and *P. xuthus* populations, determining their distribution range, and contributing to the conservation of butterfly species diversity.

The Maxent model was able to effectively identify the high distribution areas of the species. Supported by suitable habitat zones, it could provide valuable insights for butterfly conservation [38]. Understanding the relationship between species and spatial environments can improve the efficiency of species conservation practices [50]. China, as a key region for butterfly research, saw a significant decrease in the suitable habitat areas for *Papilio xuthus*, and appropriate conservation measures should have been provided. In this study, we considered 19 environmental variables and three topographical variables, though there were some limitations in practical application. As *P. xuthus* is an herbivorous insect, its distribution pattern is partly related to the distribution of its host plants [42]. Environmental factors also affect plants, indirectly influencing the distribution of *P. xuthus*. Moreover, some low-suitability areas appeared in the inland regions of Mongolia and Russia, but these regions currently do not host *P. xuthus* populations. Butterfly migration ability is also affected by environmental factors [51]. In most regions of Russia, the climate is cold, so there is almost no possibility for *P. xuthus* to migrate inland into Russia. This suggests that the Maxent model has certain limitations in considering all the factors influencing species distribution, and its predicted range may exceed the actual distribution range.

## 5. Conclusions

This study successfully utilized the Maxent model to investigate the geographic distribution and environmental adaptability of *Papilio xuthus*. The results indicated that *P. xuthus* currently has a wide distribution in East Asia, with high-suitability areas concentrated in China, Japan, North Korea, and South Korea. In these three countries, the distribution of high-suitability areas for *P. xuthus* is nearly nationwide. However, under the three future scenarios, the high-suitability areas of *P. xuthus* decreased to varying degrees, with the most significant reduction occurring in China. The proportion of area reduction within China ranged from 24.85% to 46.46%. The centroid of the high-suitability distribution shifted significantly northeast compared to the current scenario. Biological environmental factors such as Temperature Seasonality, Mean Temperature of the Wettest Quarter, Precipitation of the Wettest Month, and Precipitation of the Warmest Quarter significantly influenced the distribution of *P. xuthus*. The benefit of this study is that it provides a better understanding of the potential conservation areas for *P. xuthus* under climate change, which is of great significance for the protection of this species.

## Figures and Tables

**Table 1:** Environmental variables affecting *P. xuthus* distribution.

Code	Variable	Unit
bio04	Temperature Seasonality	C
bio08	Mean Temperature of the Wettest Quarter	°C
bio10	Mean Temperature of the Warmest Quarter	°C
bio13	Precipitation of the Wettest Month	mm
bio18	Precipitation of the Warmest Quarter	mm

**Table 2:** Potential ranges of suitable environmental variables for *P. xuthus*.

Environmental Variables	Suitable Range	Suitable Range
bio04	773.35–1054.28 °C	941.72 °C
bio08	20.11–24.42 °C	22.92 °C
bio13	179.05–368.74 mm	299.85 mm
bio18	466.25–1883.96 mm	1342.09 mm

**Table 3:** Suitable areas under current and future climate conditions.

Decade	Scenarios	Predicted Area (×10^3^ km^2^)			Comparison with Current Distribution (%)		
		Low Suitable Aera	Moderately Suitable Aera	Highly Suitable Aera	Low Suitable Aera	Moderately Suitable Area	Highly Suitable Aera
Current		2741.01	2147.53	1827.83			
2050s	Ssp126	3020.66	2803.80	1254.64	10.20 %	30.56 %	−31.36 %
	Ssp370	2550.66	3109.77	1450.80	−6.94 %	44.81 %	−20.63 %
	Ssp585	2993.66	2926.18	1531.39	9.22 %	36.26 %	−16.22 %
2090s	Ssp126	2598.47	3061.70	1555.75	−5.20 %	42.57 %	−14.89 %
	Ssp370	3966.08	2323.73	1341.44	44.69 %	8.20 %	−26.61 %
	Ssp585	3947.26	2868.35	1742.17	44.01 %	33.56 %	−4.69 %

## Data Availability

The data supporting the results are available in a public repository at: https://figshare.com/s/026d5499cb8f3f3dee06, accessed on 27 March 2024); GBIF.org GBIF Occurrence Download https://doi.org/10.15468/dl.vcz9fe, accessed on 27 March 2024).

## Supplementary Materials

The following supporting information can be downloaded at: https://www.mdpi.com/article/10.3390/insects16020131/s1, Table S1 Lists the 19 bioclimatic variables used in the modeling process, Table S2 Correlation analysis of the environmental factors used in the final model, Table S3 AUC_training_ and AUC_test_ in modeling process, Table S4 Percentage contribution and ranking of environment variables in Maxent model, Table S5 The suitable habitat area in China in the future.
