# Supplementary material for: Effects of Climate Change on the Distribution of Papilio xuthus
Source: Insects. 2025 Jan 29;16(2):131. doi: 10.3390/insects16020131 (PMC11856998; doi:10.3390/insects16020131)
Supplement: Supplementary file 1 [file insects-16-00131-s001.zip › Tables S1-S5.pdf]

Table S1 Lists the 19 bioclimatic variables used in the modeling process

Code	Environmental variables	Percent contribution(%)	Permutation importance	Unit
bio1	Annual Mean Temperature	2	5	℃
Bio2	Mean Diurnal Range	1	4	℃
Bio3	Isothermality	2.1	0.8	%
Bio4	Temperature Seasonality	14.6	3.4	℃
Bio5	Max Temperature of Warmest Month	0	0.1	℃
Bio6	Min Temperature of Coldest Month	0.6	0.1	℃
Bio7	Temperature Annual Range	0.8	0.8	℃
Bio8	Mean Temperature of Wettest Quarter	0.8	25.1	℃
Bio9	Mean Temperature of Driest Quarter	0.9	0.4	℃
bio10	Mean Temperature of Warmest Quarter	0.4	14.3	℃
bio11	Mean Temperature of Coldest Quarter	0.8	0.6	℃
bio12	Annual Precipitation	0.3	0.3	mm
bio13	Precipitation of Wettest Month	6.6	36.8	mm
bio14	Precipitation of Driest Month	0.1	0.8	mm
bio15	Precipitation Seasonality	0.5	0.9	mm
bio16	Precipitation of Wettest Quarter	0.9	1.1	mm
bio17	Precipitation of Driest Quarter	0	0	mm
bio18	Precipitation of Warmest Quarter	66.6	4.2	mm
bio19	Precipitation of Coldest Quarter	0.1	0.5	mm
elev	elevation	0.5	1	m
slope	slope	0.1	0.5	°
aspect	aspect	0	0	/

Table S2 Correlation analysis of the environmental factors used in the final model.

	bio08	bio10	bio13	bio18
bio04	-0.30	-0.35	-0.56	-0.36
bio08	0.00	0.98	0.47	0.53
bio10	0.00	0.00	0.46	0.48
bio13	0.00	0.00	0.00	0.78
bio18	0.00	0.00	0.00	0.00

Table S3 $AUC_{training}$ and AUC_{test} in modeling process.

	Repeating Group										
	1	2	3	4	5	6	7	8	9	10	Average
$AUC_{training}$	0.966	0.965	0.966	0.965	0.965	0.965	0.965	0.965	0.965	0.965	0.965
AUC_{test}	0.964	0.961	0.963	0.966	0.964	0.970	0.964	0.964	0.968	0.962	0.965

Table S4 Percentage contribution and ranking of environment variables in Maxent model.

species	Variable	Percent contribution(%)	Permutation importance
Papilio xuthus	bio18	70.3	7.5
	bio04	18.9	15.4
	Bio13	8.8	47.8

bio08	1	23.7
bio10	1	5.5

Table S5 The suitable habitat area in China in the future.

Decade	Scenarios	Predicted Area (×10^3 km^2)			Comparison with Current Distribution (%)		
		Poorly Suitable Aera	Moderately Suitable Aera	Highly Suitable Aera	Poorly Suitable Aera	Moderately Suitable Area	Highly Suitable Aera
Current		1218.26	1635.19	1326.30			
2050s	Ssp126	1591.58	2092.41	710.09	30.64%	27.96%	-46.46%
	Ssp370	1240.90	2390.90	942.10	1.86%	46.22%	-28.97%
	Ssp585	1566.22	2198.72	989.20	28.56%	34.46%	-25.42%
2090s	Ssp126	1261.53	2315.30	996.70	3.55%	41.59%	-24.85%
	Ssp370	2588.35	1621.41	744.88	112.46%	-0.84%	-43.84%
	Ssp585	2411.23	1895.50	926.41	97.92%	15.92%	-30.15%
